# Checkpoint blockade in the treatment of breast cancer: current status and future directions

**DOI:** 10.1038/s41416-018-0126-6

**Published:** 2018-05-29

**Authors:** Lironne Wein, Stephen J Luen, Peter Savas, Roberto Salgado, Sherene Loi

**Affiliations:** 1 Peter MacCallum Cancer Centre, Melbourne, VIC Australia; 2 Department of Pathology/GZA, Antwerp, Belgium; 3 University of Melbourne, Melbourne, VIC Australia

**Keywords:** Breast cancer, Cancer immunotherapy

## Abstract

There is now accumulating evidence that the host immune system plays an important role in influencing response to treatment and prognosis in breast cancer. Immunotherapy with immune checkpoint inhibitors is a promising and rapidly growing field of interest in many solid tumours, including breast cancer. Trials to date have largely focused on metastatic triple-negative disease, a genomically unstable subtype of breast cancer that is believed to be the most immunogenic and following the development of treatment resistance, has limited treatment options and a particularly poor prognosis. Both checkpoint inhibitor monotherapy and combinations with chemotherapy are being investigated. In this review, we discuss the current evidence for PD-1/PD-L1 blockade in metastatic triple-negative breast cancer (TNBC), HER2+ breast cancer and ER+ disease, as well as the emerging evidence for use in the early-stage (neoadjuvant) setting. We also propose potential ways of improving responses to checkpoint blockade in breast cancer.

## Introduction

The central role of the immune system in cancer biology and therapeutics is being increasingly recognised and studied. Breast cancer has not conventionally been considered a highly immunogenic cancer, unlike melanoma and non-small cell lung cancer, which have the highest somatic mutational load of all human cancers.^1^ Increased tumour mutational load has been reported to be associated with increased immunogenicity;^2,3^ this may be due to an increased probability of tumours with high mutational loads displaying immunogenic neoantigens, which are recognised by T cells.^4–6^ Furthermore, the immunological constant of rejection comprises a set of genes that influence the immune effector pathway,^7^ and high expression of these genes signifies an active immune environment and is associated with a favourable prognosis.^8^ However, the precise genomic determinants of immunogenicity in breast cancer remain poorly understood.

Observations of tumour infiltrating lymphocytes (TILs) in and around neoplastic cells,^9^ and the association of TILs with pathological complete response (pCR) to treatment and favourable prognosis in early breast cancer, however, have transformed our understanding of the importance of the immune system in this disease. A number of immune checkpoint inhibitors are currently in use in several types of cancer. These include antibodies against cytotoxic T lymphocyte-associated antigen 4 (CTLA-4) (ipilimumab and tremelimumab) and against the programmed death-1 (PD-1) T cell co-receptor (pembrolizumab and nivolumab) and its ligand PD-L1 (atezolizumab, avelumab and durvalumab). To date, pembrolizumab and atezolizumab are the checkpoint inhibitors that have been most extensively studied in breast cancer.

In this review, we discuss the significance of TILs in the context of conventional therapies and future immunotherapy directions. We review the evidence for immune checkpoint inhibitors in metastatic TNBC, HER2+ and ER+ disease, as well as in early-stage disease. We also discuss potential methods of improving responses to immunotherapies in breast cancer.

## TILs in breast cancer

TILs are mononuclear immune cells that are found in and around tumour tissue. The tumour microenvironment contains both pro-inflammatory immune cells and cells with immune suppressor actions. Type 1 T helper cells, cytotoxic CD8 T cells, natural killer cells, dendritic cells and M1 macrophages characterise an effective immune response, whereas M2 macrophages, myeloid-derived suppressor cells and regulatory T cells have suppressor functions.^10,11^ TILs have been observed in most solid tumour types (including breast cancer), using simple diagnostic haematoxylin and eosin (H&E)-stained slides, and they represent a surrogate for anti-tumour T cell-mediated immunity. In breast cancer, higher TIL levels are seen in triple-negative (TNBC) and HER2 positive (HER2+) tumours, compared with ER+/HER2- tumours.^12^ T cells comprise the majority of the TIL population in breast cancer,^13^ and observations of TILs in these cancers provide insight into their immunogenicity, suggesting that TNBC and HER2+ cancers are more immunogenic than ER+/HER2− cancers.^3^

There has been much interest in the use of TILs as a biomarker in breast cancer to (i) help identify patients with a favourable prognosis who may be appropriate for treatment de-escalation in the adjuvant setting, and (ii) to potentially predict responders to immune checkpoint blockade. Furthermore, we have developed the use of TILs as a biomarker using semi-quantitative scoring on H&E-stained slides, to keep this marker as simple as possible to make it widely assessable.^14,15^ The prognostic value of TILs in primary breast cancer has been substantially evaluated using tumour samples in thousands of patients from prospective trials of adjuvant chemotherapy. A strong linear relationship between increased TIL levels and improved recurrence-free survival in TNBC and HER2+ disease has been demonstrated.^12,16–18^ TILs have also been associated with higher rates of pathologic complete response (pCR) to neoadjuvant treatment.^19^ Evidence for this role is strongest in TNBC and HER2+ subtypes;^16,20–22^ in patients who have residual disease after neoadjuvant therapy, TILs in the residual disease specimen have been shown to be associated with a more favourable prognosis.^21^ Of note, increased TILs have been associated with increased rates of pCR in luminal tumours, with either no effect on survival or reduced overall survival (OS).^12,19,23^ The relationship in ER+ breast cancer is more complicated due to the association of TILs with higher proliferation rates, with the latter being associated with poorer outcomes in luminal breast cancers. More recently, the prognostic role of TILs in metastatic disease has been evaluated. It was found that in patients with advanced HER2+ breast cancer treated with docetaxel, trastuzumab and pertuzumab or placebo, higher TIL levels were significantly associated with improved OS.^24^ These data suggested that the prognostic effect of anti-tumour immunity also extends to the advanced setting.

Collectively, these results suggest that some breast cancer patients have a robust immune repertoire that may be bolstered by immunotherapy agents to achieve a robust anti-tumour response. As we will discuss below, higher response rates and longer survival have been observed in patients with higher TIL levels,^25^ which suggest that TILs may have a role as a biomarker for the identification of patients who may respond to immunotherapy. Further studies stratifying response to immunotherapy based on TIL levels in large numbers of patients are needed to establish this potential role for TILs.

## Immune checkpoint inhibitors as monotherapies in metastatic TNBC

Immunotherapy trials to date have largely been focused on patients with TNBC in the advanced disease setting. This subtype is thought to be one of the more immunogenic forms of breast cancer, at least in primary disease,^26^ and has limited treatment options apart from cytotoxic chemotherapy. Early data on the efficacy of checkpoint blockade, in particular PD-1 and PD-L1 inhibition, are just starting to emerge for breast cancer (Table 1).

**Table 1:** Immune checkpoint inhibitors in metastatic breast cancer

Study	Phase	Checkpoint inhibitor	Combination	Population	Number of patients	PD-L1 selection	ORR (RECIST 1.1)
KEYNOTE-012^27^	1b	Pembrolizumab	Nil	TNBC ; pre-treated	32	PD-L1 positive (expression in stroma or ≥1% tumour cells)	18.5%
KEYNOTE-086^29, 30^	2	Pembrolizumab	Nil	TNBC ; Cohort A, pre-treated; Cohort B, first line	Cohort A: 170 Cohort B: 52	Cohort A, unselected; Cohort B, PD-L1 positive (combined positive score ≥1%)	Cohort A: 5% Cohort B: 23%
Emens et al.^31^	1a	Atezolizumab	Nil	TNBC ; majority pre-treated	21	PD-L1 positive (≥5% of infiltrating immune cells)	24%
Schmid et al.^25^	1a	Atezolizumab	Nil	TNBC ; first line or pre-treated	115	Unselected	10%
Tolaney et al.^32^	1b/2	Pembrolizumab	Eribulin	TNBC ; first line or pre-treated	39	Unselected	33.3%
Adams et al.^34^	1b	Atezolizumab	Nab-paclitaxel	TNBC ; first line or pre-treated	32	Unselected	42%
KEYNOTE-028^36^	1b	Pembrolizumab	Nil	ER+/HER2-; pre-treated	25	PD-L1 positive (expression in stroma or ≥1% tumour cells)	12%
JAVELIN^39^	1b	Avelumab	Nil	Unselected ; pre-treated	168	Unselected	5.4%

Summary of recently presented studies of anti-PD1/PD-L1 therapy in metastatic breast cancer.

*ORR* objective response rate, *TNBC* triple-negative breast cancer

### Pembrolizumab monotherapy

KEYNOTE-012^27^ was a phase 1b trial of single-agent pembrolizumab in patients with TNBC, urothelial cancer, gastric cancer and head and neck cancer. Patients were required to be PD-L1 positive by immunohistochemistry (IHC), which was defined as PD-L1 positivity in the stroma or ≥1% tumour cells using their Qualtek assay.^28^ Most patients were heavily pre-treated; the median number of previous lines of systemic therapy for metastatic disease was two, with 46.9% of patients having received at least three lines of prior therapy and 25.0% having received at least five. Among the 32 patients in the TNBC cohort, overall response rate (ORR) was 18.5%, with median time to response of 17.9 weeks and median duration of response not yet reached (range, 15.0 to ≥47.3 weeks) at the time of reporting. Five patients (15.6%) experienced grade 3 or greater toxicity and there was one treatment-related death (disseminated intravascular coagulation). While these data were promising, it is likely that physician selection bias of patients played a part in these high response rates, because metastatic TNBC patients rarely make it past many lines of treatment, and all patients were required to be PD-L1 positive.

Phase 2 studies of pembrolizumab in metastatic TNBC have recently been reported. KEYNOTE-086 is a phase 2 trial of pembrolizumab monotherapy in metastatic TNBC as first-line or above treatment.^29,30^ Cohort A enroled 170 patients, who had received at least one prior systemic treatment for metastatic disease; 44% had three or more lines of prior therapy. ORR was 5%, with median duration of response 6.3 months (range, 1.2+ to 10.3+). Grade 3 or 4 treatment-related adverse events occurred in 12% of patients, and there were no deaths due to adverse events. Cohort B included patients with previously untreated metastatic disease, with a tumour PD-L1 combined positive score ≥1%. Data from the first 52 patients enroled showed an ORR of 23%, with 4% achieving a complete response. Of note, there were few objective responses observed with liver metastases, visceral disease, higher LDH and higher tumour burden; however, this observation needs further validation in larger cohorts. Importantly, the objective responses seen were durable. The safety profile was very manageable, with five reported grade 3 or 4 treatment-related adverse events, and no patients dying or discontinuing due to an adverse event. These data suggested that the first-line setting was the best time to evaluate pembrolizumab monotherapy. We speculate that the lower responses after the first-line setting likely relates to a larger tumour burden (particularly in liver disease), selection of the less immunogenic tumour clones and the high growth rate of disease in advanced TNBC. Biomarkers are clearly needed in this setting, because the chance of a durable response with pembrolizumab monotherapy without cytotoxic chemotherapy is appealing for the patients that can derive this benefit. Phase 3 studies will be necessary to more fully evaluate the efficacy of pembrolizumab. KEYNOTE-119 (NCT02555657) is an ongoing phase 3 trial of single-agent pembrolizumab versus single-agent chemotherapy of physician’s choice for metastatic TNBC. Patients have been pre-treated with 1 or 2 prior lines of therapy in the metastatic setting. This trial has finished recruitment and is expected to report in 2019.

### Atezolizumab monotherapy

The anti-PD-L1 antibody atezolizumab has also been investigated in early phase trials in metastatic breast cancer. In a phase 1a study of atezolizumab in metastatic TNBC,^31^ 21 patents with PD-L1 positive (≥5% of infiltrating immune cells) disease were evaluated for efficacy. ORR was 24%, including two complete responses. Duration of response ranged from 0.1 to 41.6 weeks, with the median not yet reached. Grade 3–5 adverse events were experienced by 11% of patients, and one death was reported from pulmonary hypertension. In addition, Schmid et al. recently reported the activity of atezolizumab monotherapy in a metastatic TNBC cohort of a Phase Ia study.^25^ Most patients were heavily pre-treated in this cohort. Eleven percent of patients experienced grade 3–4 treatment-related adverse events, and 2% experienced a grade 5 treatment-related adverse event. Interestingly, while ORR was only 10%, all of these responding patients achieved long-term survival benefit (2-year OS 100%). This is notable, given the median OS of advanced TNBC is around 1 year. Higher ORRs were observed in those who were being treated first line and also those with PD-L1 ≥5%. Higher levels of TILs (evaluated as median TIL infiltration as a percentage of tumour area) were also associated with higher ORRs and longer OS; in the group with TILs ≤10%, median OS was 6.6 months, compared with 12.6 months for those with TILs >10%.

Considering these details, it may be that a combination of TILs on H&E and PD-L1 IHC may be optimal in identifying patients who will respond to checkpoint blockade. Furthermore, it is notable that ORRs seen with atezolizumab and pembrolizumab are similar, suggesting comparable efficacy of these PD-1 and PD-L1 inhibitors in breast cancer.

## Immune checkpoint inhibitors as combination therapies in metastatic TNBC

Due to the modest responses observed with monotherapy, combinations of PD-1/PD-L1 blockade and chemotherapy are being studied. Tolaney et al. conducted a phase 1b/2 study to evaluate the safety and efficacy of eribulin combined with pembrolizumab in metastatic TNBC.^32^ Patients had received two or fewer lines of previous chemotherapy in the metastatic setting. ORR was 33.3% and 7.7% of patients achieved durable stable disease, defined as stable disease with a duration of 24 weeks or more after first dose date. Two-thirds of patients experienced grade 3 or 4 treatment-emergent adverse events, no treatment-related deaths occurred. Possible immune-related grade 3 or 4 events occurred in 12.8% of patients and included rash, hyperglycaemia, pneumonitis and renal failure. The efficacy of pembrolizumab combined with chemotherapy is being further investigated in large clinical studies. For example, KEYNOTE-355 (NCT02819518), which is a phase 3 study, is investigating the safety and efficacy of pembrolizumab plus chemotherapy. This combination will be compared to placebo plus chemotherapy as first-line treatment for advanced TNBC. The pembrolizumab dose in these trials is 200 mg every 3 weeks and patients are unselected for PD-L1 positivity. This study is currently recruiting, and is expected to be complete in early 2019.

Combination regimens with chemotherapy/atezolizumab are also being investigated. In a study combining atezolizumab with nab-paclitaxel in advanced TNBC, an ORR of 42% was reported.^33,34^ Eleven of 17 responses (65%) were continuing at the time of data cutoff. After a median safety follow-up of 5.2 months (range, 0.6–12.6), five patients had discontinued nab-paclitaxel due to an adverse event, and no treatment-related deaths were observed. Based on these results, IMpassion130 (NCT02425891) was commenced. This is a phase 3 randomised trial of atezolizumab in combination with nab-paclitaxel compared with placebo plus nab-paclitaxel in previously untreated metastatic TNBC. Recruitment has finished recruitment and the trial is likely to report in 2018.

## Immune checkpoint inhibitors in HER2+ and ER+ subtypes

High levels of immune infiltrate have also been observed in early-stage HER2+ breast cancers, and associations with prognosis and prediction have been reported.^12,16,19,22,24^ Immunotherapy is therefore currently being investigated in the setting of metastatic HER2+ disease. PANACEA (NCT02129556; KEYNOTE-014) is a phase 1b/2 trial of pembrolizumab in advanced, trastuzumab-resistant, HER2+ breast cancer. In this study, different dose levels of pembrolizumab (1 mg/kg, 2 mg/kg, 10 mg/kg or a flat dose of 200 mg every 3 weeks) combined with trastuzumab was  given to patients who have progressed on prior trastuzumab, or recurred while on adjuvant trastuzumab or within 1 year of completing adjuvant trastuzumab. The combination of trastuzumab emtansine (TDM-1) and atezolizumab is being investigated in the KATE2 trial (NCT02924883). TDM-1 has been shown in preclinical models to promote anti-tumour immunity.^35^ This is a phase 2 study investigating the safety and efficacy of this treatment in patients who have received prior trastuzumab and taxane-based therapy. Furthermore, several other studies of combined HER2-targeted treatment with immune checkpoint blockade are currently recruiting (e.g., NCT02318901 and NCT02605915).

There are currently little data regarding efficacy in ER+ breast cancers. KEYNOTE-028 is an ongoing phase 1b study evaluating the efficacy and safety of pembrolizumab in patients with PD-L1 positive (expression in stroma or ≥1% tumour cells) advanced solid tumours.^36^ The breast cancer cohort comprises 25 patients with ER+/HER2− tumours, a breast cancer subtype generally considered to be less immunogenic than TNBC or HER2+ disease. Only 19% of the 248 patients screened had PD-L1 positive tumours, suggesting low immunogenicity in this cohort. Patients were heavily pre-treated, with nearly half of the patients having received five or more previous lines of treatment for advanced disease. Three patients (12%) achieved a partial response, with the median duration of response not yet reached (range, 8.7 to 44.3 weeks). Overall, 16% of patients experienced a grade 3 or 4 treatment-related adverse event, and there were no treatment-related deaths. Of note, oestrogen has been shown to induce the mobilisation of myeloid-derived suppressor cells and enhance their immunosuppressive activity.^37^ Therefore, oestrogen may have immunosuppressive effects in the microenvironment that may also affect responses to immunotherapies^37,38^; this may, at least partly, explain the low response rate to pembrolizumab seen in the KEYNOTE-028 cohort.

In the metastatic breast cancer expansion cohort of the JAVELIN study,^39^ the PD-L1 antibody avelumab was used in 168 patients with metastatic breast cancer, unselected by subtype or PD-L1 status. Patients had been treated with a median of three prior lines of therapy in the locally advanced or metastatic setting. ORR was 5.4%, with one complete response and eight partial responses, and five of nine responses were still ongoing at the time of cutoff. Stable disease was seen in an additional 23.8% of patients. Notably, for patients with PD-L1 positive immune cells within the tumour, 33.3% had partial responses. Treatment-related grade 3 or higher adverse events were seen in 14.3% of patients, with two treatment-related deaths having been recorded (one acute liver failure and one respiratory distress).

Collectively, these results indicate that the efficacy of immunotherapy in metastatic breast cancer outside of the TNBC setting is in the very early stages of investigation, and much work is needed to establish whether there is a role for these agents for HER2+ and ER+ disease.

## Immune checkpoint inhibitors in the neoadjuvant setting

Neoadjuvant therapy is used in breast cancer to downstage tumours and eliminate micrometastases. Much emphasis is placed on achieving a pCR, as this is highly correlated with long-term outcome^40^ in TNBC and HER2+ disease, and trials are currently being conducted to investigate the use of checkpoint inhibitors in this setting (Table 2).

**Table 2:** Immune checkpoint inhibitors in primary breast cancer

Study	Phase	Checkpoint inhibitor	Chemotherapy	Population	No. of patients	pCR (ypT0/is and ypN0)
I-SPY 2^43^	2	Pembrolizumab	Paclitaxel or paclitaxel/pembro followed by doxorubicin/cyclophosphamide	TNBC ; HR+/HER2- ; PD-L1 unselected ; tumour size > 2.5 cm; mammaprint high risk (nodal involvement in 37.7% pembro, 43.9% control)	69 pembro, 180 control	TNBC: 60% pembro vs. 20% control^a^ HR+/HER2-: 34% pembro vs. 13% control^a^
KEYNOTE-173^41^	1b	Pembrolizumab	A: pembro followed by pembro + nab-paclitaxel followed by pembro + doxorubicin/cyclophosphamide . B: pembro followed by pembro + nab-paclitaxel + carboplatin followed by pembro + doxorubicin/cyclophosphamide	TNBC ; PD-L1 unselected ; locally advanced (primary tumour stage ≥T2 in 90%, nodal involvement in 75%)	20	Cohort A, 60%; Cohort B, 90%
Pusztai et al.^42^	1	MEDI4736	MEDI4736 + nab-paclitaxel followed by dose dense doxorubicin/cyclophosphamide	TNBC ; PD-L1 unselected; stage I-III (primary tumour stage ≥T2 in 57%, nodal involvement in 57%)	7	71.4%

Recently presented studies of anti-PD1/PD-L1 agents in neoadjuvant breast cancer therapy.

*pCR* pathological complete response.

^a^Estimated pCR

The phase Ib KEYNOTE-173 study (NCT02622074) evaluated pembrolizumab plus chemotherapy as neoadjuvant therapy for locally advanced TNBC. Preliminary data from 20 patients showed an impressive pCR rate of 50% in cohort A (pembrolizumab plus nab-paclitaxel followed by pembrolizumab plus AC [doxorubicin plus cyclophosphamide]) and 90% in cohort B (pembrolizumab plus nab-paclitaxel plus carboplatin followed by pembrolizumab plus AC).^41^ Grade 3–4 treatment-related adverse events occurred in eight patients in cohort A and ten patients in cohort B. One patient in A and two patients in B discontinued for a treatment-related adverse event (two ALT elevations with pembrolizumab; one DVT with chemotherapy). Pusztai et al. evaluated the safety of the anti-PD-L1 antibody MEDI4736 administered concomitantly with neoadjuvant chemotherapy for TNBC.^42^ MEDI4736 was administered with weekly nab-paclitaxel and subsequently with dose-dense AC. Three patients completed therapy at the 3 mg/kg dose without any dose-limiting toxicities. At the 10 mg/kg dose level, all three patients completed the nab-paclitaxel + MEDI4736 treatment without any dose-limiting toxicities. The phase 2 portion of the trial is currently open to recruitment (NCT02489448).

I-SPY 2 is phase 2 platform which is evaluating novel neoadjuvant agents on a backbone of weekly paclitaxel followed by AC, and selected by Mammaprint high-risk score (NCT01042379). Bayesian models are being used to estimate the pCR rates. Nanda et al. investigated the addition of pembrolizumab to standard neoadjuvant therapy, where they reported a tripling the estimated pCR rate in TNBC (60% vs. 20%), and near tripling of the estimated pCR rate in hormone receptor+/HER2− (34% vs. 13%).^43^ Of note, five patients (7%) experienced grade 3 immune-related adverse events (one hypophysitis and four adrenal insufficiency). Pembrolizumab was given upfront with the weekly paclitaxel and not continued with the AC component.

The apparent high rate of immune-related adverse events seen with the I-SPY 2 study is intriguing. It could be that the immune system is more robust and could be easier to modulate in the early-stage setting and/or it may be that the chemotherapy given in the KEYNOTE-173 study resulted in significant leukopenia, which may have impacted the immune response. However, the high response rates seen suggest that early-stage disease may be the preferable setting for immunotherapy (as evidenced by high levels of TILs in primary tumours). This will require well thought out clinical trials to avoid over treatment. Indeed, it could be that pCR may not be the best end point, as it is likely that the patients with high TILs will benefit from PD-1 blockade, and one could speculate that many of these patients may have done well with chemotherapy alone despite not achieving pCR. An early on-treatment biopsy to understand if patients with low levels of immune infiltrate at baseline can increase their immune response might be one way of investigating whether immunotherapy is beneficial to those who really need additional treatment in the early-stage setting.

Beyond the neoadjuvant setting, trials are also being conducted to assess the efficacy of immune checkpoint inhibitors used as post neoadjuvant therapy in TNBC. Phase 3 trials of atezolizumab and pembrolizumab are both currently underway (NCT03281954 and NCT03036488).

## Improving responses to immunotherapy in breast cancer

**Fig. 1:**
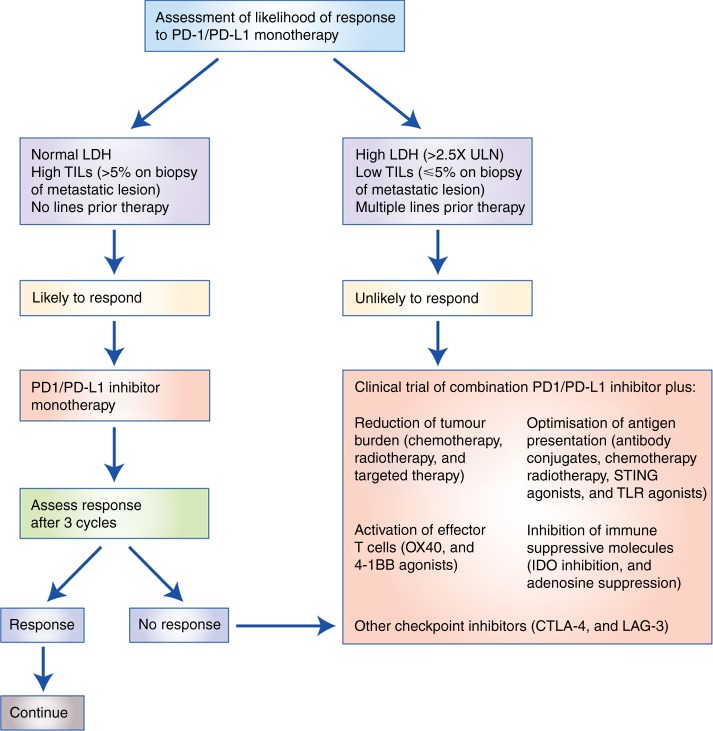
Proposed schema for treating metastatic TNBC with PD-1/PD-L1 blockade. Based on findings presented by Loi et al.^64^ CTLA-4 cytotoxic T lymphocyte-associated protein 4, IDO indoleamine 2,3-dioxygenase, LAG-3 lymphocyte-activation gene 3, LDH lactate dehydrogenase, TIGIT T cell immunoreceptor with Ig and ITIM domains, TILs tumour infiltrating lymphocytes, TLR toll-like receptors, STING stimulator of interferon genes, ULN upper limit of normal

Observed response rates to immunotherapy in breast cancer are modest compared with some other tumours, such as melanoma and non-small cell lung cancer. This may be due to well-evolved tumour immune escape mechanisms, such as a reduced expression of major histocompatibility complex class I (MHC I), resulting in decreased immune recognition,^11^ and an immunosuppressive tumour microenvironment present in advanced disease.^44^ However, it is clear that the minority of patients who respond to PD-1/PD-L1 blockade can derive substantial benefit and that the responses can be durable. This is notable because a minority of advanced breast cancer patients may have durable tumour control without chemotherapy; the key will be in identifying a biomarker that can identify these patients. There are several potential approaches that may be taken to improve these response rates (Fig. 1).

### Combination therapy

Combining chemotherapy, targeted therapy or radiotherapy with immunotherapy may enhance responses by increasing immunogenicity or overcoming mechanisms of immune escape. There is growing evidence that response to conventional anticancer agents is mediated, at least in part, by the immune system^45,46^ and the combination of immune checkpoint inhibitors with chemotherapy, targeted therapy and/or radiotherapy may be synergistic.^47,48^ As described above, current multimodality combinations being investigated include pembrolizumab plus either trastuzumab or eribulin, and atezolizumab plus either nab-paclitaxel or TDM-1. In addition, trials are currently underway with immune checkpoint inhibitors combined with a MEK inhibitor/chemotherapy (NCT02322814) and with radiotherapy (NCT02303366).

Another combinatorial approach involves dual checkpoint inhibition. In melanoma, combined PD-1 and CTLA-4 inhibition, while associated with significant toxicity, has been shown to be effective.^49^ Such combinations are yet to be adequately investigated in breast cancer (NCT02892734), as are other checkpoint proteins such as LAG-3.^50^ Another potential way to target anti-PD-1 resistant tumours may be via indoleamine 2,3-dioxygenase 1 (IDO1) inhibition,^51^ or by targeting the adensoine pathway^52^ (NCT02655822). In a mouse model of anti-PD1 resistance, TILs were found to overexpress IDO1, and IDO inhibition was effective in reducing both tumour growth and lung metastasis.^53^ Certainly in melanoma, adding an IDO pathway inhibitor to a checkpoint inhibitor seems to be a promising strategy.^54^ Other strategies for further investigation include (1) optimisation of antigen presentation using antibody conjugates such as sacitizimab govitecan (IMMU-132)^55,56^ and STING or TLR agonists,^57^ and (2) use of agents which activate effector T cells such as OX40 agonists^58^ and 4-1BB agonists.^59^

### Patient selection using biomarkers

Patients may be better selected for immunotherapy using an accurate predictive biomarker, such as PD-L1 expression and TILs (Table 3). There is some evidence that PD-L1 positivity is associated with response to immunotherapy in breast cancer;^60^ however, use of PD-L1 expression as a biomarker is limited by poor standardisation of assays and cutoffs. In addition, some patients with PD-L1 negative tumours have been noted to respond to anti-PD-1/PD-L1-directed treatments, with some of these responses being durable, thus limiting PD-L1 expression as an exclusionary biomarker.^61^ Evaluation of PD-L1 protein levels on tumour samples has been fraught with difficulties with regard to assay types and reproducibility. TILs as seen by H&E are indicators of tumour immunogenicity, and have demonstrated an association with response to neoadjuvant therapy and trastuzumab-based therapy.^16,19^ The clinical utility of TILs to predict response to immunotherapy in the metastatic setting, while biologically plausible, has yet to be adequately explored. Much work remains to be done with regard to appropriate biomarkers in this space.

**Table 3:** Potential biomarkers for patient selection for immunotherapy in breast cancer

Biomarker	Method of assessment	Evidence
TILs	H&E slides	Pembrolizumab monotherapy in metastatic TNBC: Median (IQR) TIL level in responders vs. non-responders was 10% (7.5–25%) vs. 5% (1–10%) in cohort A (previously treated, any PD-L1 expression) and 50% (5–70%) vs. 15% (5–37.5%) in cohort B (previously untreated, PD-L1 positive)^64^
PDL1	IHC (VENTANA PD-L1 (SP142) Assay)	Atezolizumab in combination with nab-paclitaxel in metastatic TNBC: ORR IC0 = 57.1% (95% CI: 18.4, 90.1) ; ORR IC1/2/3 = 77.8% (95% CI: 40.0, 97.2);^60^ IC0 = <1% of immune cells or tumour cells staining positive for PD-L1 ; IC1 = ≥1% and <5% of immune cells or tumour cells staining positive for PD-L1 ; IC2 = ≥5% and <10% of immune cells or tumour cells staining positive for PD-L1 ; IC3 = ≥10% of immune cells or tumour cells staining positive for PD-L1

*H&E* haematoxylin and eosin, *IHC* immunohistochemistry, *IQR* interquartile range

### Immunotherapy in the first-line setting

The observation that metastatic TNBC tumours have fewer TILs than their matched primary tumours suggests that immune suppression becomes more prevalent with rapidly increasing growth, large tumour burden, and metastasis.^62^ Chemotherapy-induced lymphopenia may also be a reason for lower responses to checkpoint blockade seen in later lines of therapy. Immune microenvironment heterogeneity by organ site may also be relevant, with far fewer responses observed in patients with liver metastases.^29^ We hypothesise that immunotherapy may be more effective when used in early-stage disease and as first-line treatment in the metastatic setting, rather than in more advanced disease when host anti-tumour immune responses are diminished. In the study by Adams et al. investigating the atezolizumab/nab-paclitaxel combination,^60^ higher response rates were seen in patients who received this treatment as first-line therapy compared with second-line therapy (confirmed ORR 66.7% vs. 25%, respectively). In the advanced setting, with high tumour burden and rapidly growing disease, more aggressive approaches to re-instate immunity, such as CAR-T cells or T cell biospecifics, may be required.^63^

## Conclusion

Immunotherapy is a promising treatment approach for metastatic TNBC, and particularly PD-1/PD-L1 blockade, with phase 1 and 2 trials reporting response rates between 5 and 42%. It is likely that immunotherapy will also prove useful in HER2+ metastatic breast cancer, and in a subset of patients with early-stage disease. Trials are currently underway to establish and define the role of checkpoint inhibitors in breast cancer in each of these settings. Response rates might be improved by combining checkpoint inhibitors with chemotherapy, radiotherapy, targeted therapy, MEK inhibition, adenosine suppression or IDO inhibition, and this is a key direction for future clinical trials. Furthermore, the appropriate selection of patients for immunotherapies using biomarkers, such as TILs and PD-L1 expression, is an area of great interest and active investigation.
